# Strong Deep-Level-Emission Photoluminescence in NiO Nanoparticles

**DOI:** 10.3390/nano7080231

**Published:** 2017-08-22

**Authors:** Ashish Chhaganlal Gandhi, Sheng Yun Wu

**Affiliations:** 1 Department of Physics, National Dong Hwa University, Hualien 97401, Taiwan; acg.gandhi@gmail.com; 2 Center for Condensed Matter Sciences, National Taiwan University, Taipei 10617, Taiwan

**Keywords:** NiO nanoparticles, nickel/oxygen vacancies, interstitial oxygen, photoluminescence, electron density

## Abstract

Nickel oxide is one of the highly promising semiconducting materials, but its large band gap (3.7 to 4 eV) limits its use in practical applications. Here we report the effect of nickel/oxygen vacancies and interstitial defects on the near-band-edge (NBE) and deep-level-emission (DLE) in various sizes of nickel oxide (NiO) nanoparticles. The ultraviolet (UV) emission originated from excitonic recombination corresponding near-band-edge (NBE) transition of NiO, while deep-level-emission (DLE) in the visible region due to various structural defects such as oxygen vacancies and interstitial defects. We found that the NiO nanoparticles exhibit a strong green band emission around ~2.37 eV in all samples, covering 80% integrated intensity of PL spectra. This apparently anomalous phenomenon is attributed to photogenerated holes trapped in the deep level oxygen vacancy recombining with the electrons trapped in a shallow level located just below the conducting band.

## 1. Introduction

The wide band gap of metal oxide semiconductors has become an attractive topic from both a fundamental and a technological point of view [1]. Particularly at the nanoscale, varied and improved properties of metal oxides originate from a large surface to volume ratio of atoms, spatial confinement, reduced dimensions, lattice imperfections, and point defects (such as cation or oxygen ion vacancies) [2]. Most of the metal oxide semiconductors are limited to the harmful and expensive UV region (1.24 to 3.26 eV). However, the properties of metal oxide semiconductors can be tailored by introducing defects such that the band gap energy can narrow down to the visible region [3,4,5,6,7,8,9,10]. In recent years, the oxides of transition metals have been explored for multiple technological applications such as solar cells, catalysis, varistors, supercapacitors, gas sensors, magnetic storage, electrochromic devices, etc. [3]. Among the oxides of transition metals, nickel oxide (NiO), which is an antiferromagnetic insulator, shows *p*-type semiconducting properties, and possesses a wide band gap of 3.6–4.0 eV, has been proven to exhibit enhanced optical, magnetic, and electrical properties at the nanoscale because of nickel/oxygen vacancy defects, the easiest point defects to be formed in nanoparticles [11,12,13,14,15,16,17]. Due to its low cost and wide application, NiO is an attractive material from both a fundamental and an application point of view. In the last decades, numerous application-oriented studies such as smart windows, *p*-type transparent conducting films, chemical gas sensors, catalysts in fuel cell electrodes, electrochemical devices, and solar thermal absorbers have been carried out, which proves the potential of NiO compared to other transition metal oxides [4,18,19,20].

Photoluminescence (PL) spectroscopy is a powerful tool to characterize the optical quality of semiconductor metal oxides as PL intensity can be correlated directly with the defect densities [12]. Therefore, the PL spectra of such metal oxides are strongly affected by cationic/anionic vacancies. This can give insight into the charge excitation, electronic structure, and defect states of oxides. Recently Madhu et al. [12] reported a narrow band gap in NiO nanoparticles (size, 32–45 nm, synthesize using chemical route) extending from the UV to the visible region (3.26 to 2.53 eV). The relative Ni^2+^ and O^2−^ vacancy concentrations influence the UV-visible absorption spectra. The authors claimed that the oxygen vacancies formed in small NiO nanoparticles suppress the low energy (2–4 eV) intra-ionic 3d^8^-3d^8*^ transitions. Annealing the same sources of samples in an oxygen atmosphere at a higher temperature (forming large particles) results in the suppression of oxygen vacancies and an increase in Ni^2+^ vacancies, which results in an enhancement of these transitions. However, from our previous work and plenty of reports from other research groups, we know that the decrease of nickel vacancy concentration along with interstitial oxygen is accompanied by an increase in the NiO annealing temperature (and so the nanoparticle size) [21]. According to Naday et al. [22,23], non-stoichiometric NiO with interstitial oxygen contained many Ni^2+^ vacancies, and to preserve the charge neutrality some of the Ni^2+^ were oxidized to Ni^3+^, resulting in *p*-type conductivity. An increase in nickel vacancy defects resulted in a decrease in the main band gap energy [12]. Also, there are reports claiming broad, intense green-band emission spectra from NiO nanoparticles due to nickel vacancies [16]. However, so far the intense green-band emissions, which are generally assigned to oxygen vacancy defects, have not been discovered in NiO nanoparticles. Recently, Børseth et al. [24] utilized PL spectroscopy to identify oxygen and zinc vacancy optical single crystallite ZnO samples annealed in Zn-rich and O-rich atmosphere. The author attributed green-band emissions at 2.53 ± 0.05 eV to oxygen vacancies (former) and 2.35 ± 0.05 eV to zinc vacancies (later). Therefore, based on the above claim, thorough work needs to be carried out to understand the effect of Ni^2+^/O^2−^ vacancies and/or interstitial oxygen on the PL spectra of NiO nanoparticles.

In this work, the effect of crystallite size reduction on the electron density distribution, along with its consequences for the structural and optical properties of chemically synthesized NiO nanoparticles, are studied via the Rietveld refined XRD spectra and photoluminescence spectroscopy, respectively. This study throws further light on the effect of nickel vacancies in the surface, interstitial oxygen, and nickel and the oxygen-vacancy defects on the band gap energy in NiO nanoparticles with a crystallite size varying from 16.6 ± 0.7 nm to 54 ± 6 nm.

## 2. Synthesis of NiO Nanoparticles

A detailed structural and morphological analysis of NiO nanoparticles synthesized through the chemical route using the sol-gel method is in our previous report [21]. The calculated crystallite size of the polycrystalline NiO nanoparticle obtained after annealing in an ambient atmosphere for a time period of 1 h at T_A_ = 400 °C, 500 °C, 600 °C, 700 °C, and 800 °C using synchrotron radiation XRD spectra by the Williamson–Hall method is 16.6 ± 0.7 nm, 19.5 ± 0.6 nm, 29 ± 4 nm, 31 ± 1 nm and 54 ± 6 nm, respectively. In our previous work confocal Raman spectroscopy has been successfully utilized to probe the superexchange interaction energy of two magnon (*E_2M_*) along the next-nearest-neighbor (NNN) Ni^2+^ ions through oxygen [21]. Raman spectroscopy is surface-sensitive and therefore the observed increasing behavior of *E_2M_* with increasing nanoparticle size was attributed to the decrease in Ni vacancy concentration at the surface. The intensity of LO mode decreased relative to that of the 2LO mode with the increase in crystallite size. First-order TO and LO modes originated from the parity-breaking defects, which can be confirmed by the observed enhanced intensity for the LO mode from the black NiO nanoparticles, having the highest percentage of nickel vacancy concentration [16]. The Ni/O atomic percentage estimated from energy-dispersive spectroscopy (EDS) varies from 79% to 94.4%, approaching the stoichiometric value of 100% with an increase in the annealing temperature. Furthermore, the isothermal magnetic measurement, M(*H_a_*) of all these samples was carried out below blocking temperature. The spontaneous exchange bias (SEB) phenomenon was observed from small NiO nanoparticles [25]. The nanoparticles having nickel vacancies on the surface when exposed to an external field form short-range ordered clusters of spins that behave like weak ferromagnetic (FM) domain. The intercoupling between weak FM and uncompensated antiferromagnetic core resulted in an SEB field. Added to that, M(*Ha*) loop has shown two-component behavior, a ferromagnetic component in the low field region from short-range ordered FM clusters and antiferromagnetic, non-saturating behavior in the high field region. Therefore, in the first place our findings suggest the presence of the highest amount of nickel vacancy concentration on the surface of the smallest nanoparticles. Such small NiO nanoparticles act as a core/shell such that the amount of nickel vacancies distributed on the shell is higher than that in the core, as confirmed through magnetic, Raman, and X-ray absorption near edge spectroscopy (XANES) [26]. To study the effect of nickel/oxygen vacancies and excess interstitial oxygen defects on the band gap and trap levels of chemically synthesized, various sized NiO nanoparticles, in the powder forms were excited by a 266 nm wavelength laser. The room-temperature PL spectra of various sizes of NiO nanoparticle powder samples were detected from 250 nm to 700 nm by a QE65000 charge-coupled device imaging spectrometer. A Q-switched diode-pumped solid-state laser (266 nm) acted as the pumping light source.

## 3. Results and Discussion

### 3.1. Electron Density Analysis

**Figure 1:**
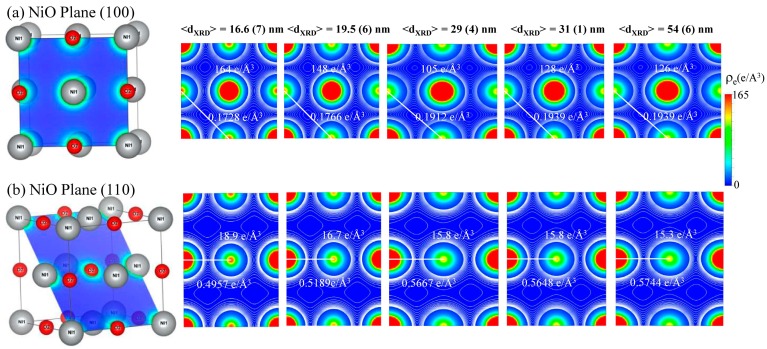
Two-dimensional electron density maps drawn (**a**) parallel to (100) and (**b**) (110) crystallographic planes of NiO nanoparticles with size ranging from 16.6 ± 0.7 nm to 54 ± 6 nm (left to right), respectively.

**Figure 2:**
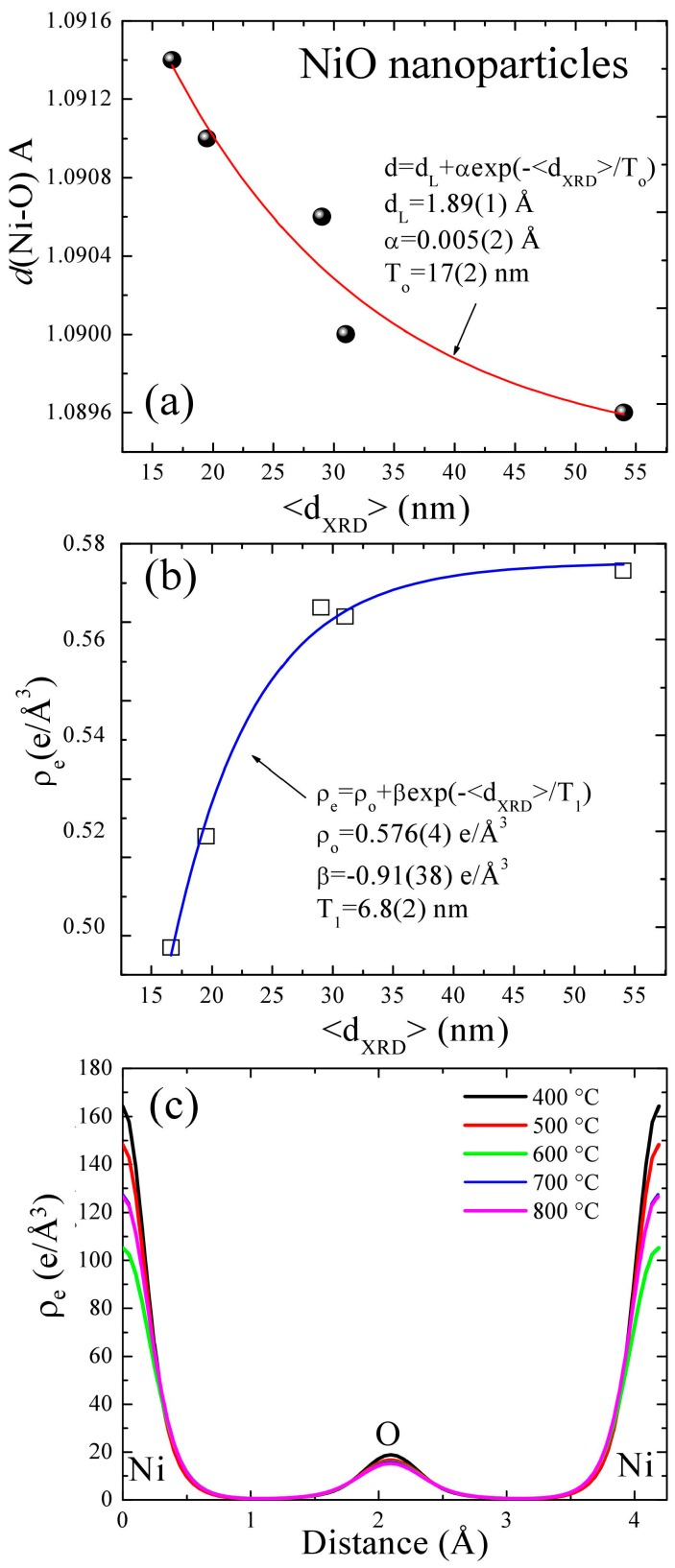
(**a**) Plot of the particle size dependence of the Ni–O bond length and (**b**) electron charge density *ρ_e_* at the critical point of Ni–O; (**c**) a plot of the one-dimensional charge density profile drawn between the Ni and O atoms at various annealing temperatures.

In the present work, the VESTA software package [27] is used to calculate the electron density distribution from the structural parameters and atomic scattering factors of NiO nanoparticles obtained by refining XRD spectra via Rietveld analysis. The effect of 1h annealing at T_A_ = 400 °C to 800 °C resulted in the color change of NiO powder samples from black to green due to a decrease in nickel vacancies with an increase of crystallite size from 16.6 ± 0.7 nm to 54 ± 6 nm [28,29], indicating that small nanoparticles are highly non-stoichiometric [30]. The structural analysis of NiO nanoparticles was carried out by refining synchrotron radiation XRD spectra using Rietveld refinement using the GSAS software package [21]. As nanoparticles were synthesized by annealing the source sample at several different temperatures, the thermal decomposition was found to play an important role in defining the stoichiometry of the nanoparticles in response to annealing temperature. A lattice expansion, having a lattice constant of 4.1900 ± 0.0004 Å, was observed from small nanoparticles, 16.6 ± 0.7 nm, T_A_ = 400 °C. Further thermal annealing resulted in an increase in crystallite size and subsequently a lattice parameter approach to the bulk value of 4.1800 ± 0.0001 Å from T_A_ = 800 °C, 54 ± 6 nm NiO nanoparticles. A similar kind of lattice expansion has been reported from small NiO nanoparticles [31] and has been attributed to the presence of interstitial oxygen and cation/anion vacancies [11]. The Ni vacancies in some of the regular lattice sites result in a reduction in density (6.596 g/cm^3^ (54 ± 6 nm) to 6.123 g/cm^3^ (16.6 ± 0.7 nm)) compared to bulk value 6.67 g/cm^3^ in small particles. The face-centered cubic (*fcc*) unit cell of NiO with a Ni atom at (0, 0, 0) position and O atom at (1/2, 1/2, 1/2) position, having a lattice constant of 4.1710 Å (bulk) and space group of *Fm-3m* (No. 225), is shown in Figure 1a. The isosurface of electron density distribution determined by Fourier transform of structure factors, *F*(*s*) is calculated from structure parameters and atomic scattering factors of free atoms [32] obtained after performing Rietveld refinement using GSAS software of XRD spectra in VEST software. The electron density, *ρ_e_*(*x*, *y*, *z*), at the coordinates of (*x*, *y*, *z*) is calculated by,

(1) $$\rho_{e}\left(x,\;y,\;z\right)=\frac{1}{V}\sum_{h=\frac{-N_{x}}{2}}^{\frac{N_{x}}{2}}\sum_{k=\frac{-N_{y}}{2}}^{\frac{N_{y}}{2}}\sum_{l=\frac{-N_{z}}{2}}^{\frac{N_{z}}{2}}F\left(s\right)exp\left[-2\pi i\left(hx+ky+lz\right)\right],\;$$

where *N* is the number of grids along each crystallographic axis and gives a resolution close to the specified value, and *V* is the volume of the unit cell. Figure 1a,b presents two-dimensional (2D) electron density maps corresponding to the increase in crystallite size from left to right. The demonstrated planes are parallel to the (100) (Figure 1a) and (110) (Figure 1b) crystallographic planes, situated on the plane spacing of 0 and 1 × *d*, respectively. The (110) plane is situated at a distance of 2.9623, 2.9615, 2.9609, 2.9574, and 2.9574 Å from the origin of the primitive cell, left to right, respectively. The counter color lines are drawn from 0 to 165 e/A^−3^ at 5 e/A^3^ intervals. The corresponding Ni–O bond length d(Ni–O) versus the crystallite size <d_XRD_> of the NiO nanoparticles are shown in Figure 2a, revealing an increase with the decrease in the crystallite size <d_XRD_>. The red solid curve indicates the fit of the data to the theoretical curve for an exponential function, d(Ni–O) = d_L_ + αexp(–<d_XRD_>/T_O_), where d_L_ = 1.089(1) Å, α = 0.005(2) Å, and T_O_ = 17(2) nm represents the initial constant and the fitted parameters, respectively. A slight lattice expansion of 0.184% (percent deviation) was observed from the <d_XRD_> = 16.6 ± 0.7 nm sample and as the crystallite size <d_XRD_> decreased further, it approaches the bulk value of ~1.0896 Å, as shown in Figure 2a. The shift is due to a decrease in nickel vacancy concentration as the system approaches stoichiometric NiO with the increase in crystallite size. The 2D maps reveal that mid-bond electron density *ρ_e_* of Ni–O bond decreases from 0.5744 Å^−3^ to 0.4957 Å^−3^ with the decrease in crystallite size <d_XRD_> due to the symmetric nature of the cubic NiO nanostructure, as can be seen in Figure 2c. The blue solid curve indicates the fit of the data to the theoretical curve for an exponential function, *ρ*_e_ = *ρ*_o_ + βexp(–<d_XRD_>/T_1_), where *ρ*_o_ = 0.576(4) e/Å^3^, β = −0.91(38) e/Å^3^, and T_1_ = 6.8(2) nm represents the initial constant and the fitted parameters, respectively. Simultaneously, a mid-bond electron density *ρ_e_* shifts toward the Ni-atom from 1.0914 Å to 1.0896 Å, which is clearly visible in the one-dimensional charge density profile drawn between the Ni and O atoms, as shown in Figure 2c (one-dimensional electron density *ρ_e_* distribution along the (100) direction). The 2D Miller maps show the perfectly polar/ionic nature of the Ni–O bond. To search for further quantitative understanding of this effect on the O–O bond, electron density distribution analysis methods were also carried out by calculating the d(O–O) bond length and *ρ*_e_ electron density. The O–O bond also strengthens with annealing temperature, as is evident from the increase in mid-bond electron density from 0.1728 e/Å^3^ to 0.1939 e/Å^3^ with crystallite size, which is a further indication of a reduction in interstitial oxygen due to the annealing effect. The values of electron density at bond critical points of all the samples are presented in Table 1, which confirm and quantify the increase in covalent strength of the bond with the crystallite size. With an increase in the annealing temperature, the concentration of both the oxygen vacancies and the interstitial oxygen decreased, providing a green stoichiometric NiO nanopowder. The values of charge density at the Ni and O core are also tabulated in Table 1, showing a maximum value of 164 and 18.9 e/Å^3^, respectively, for the smallest (19.5 ± 0.6 nm) NiO nanoparticles.

### 3.2. EDS Analysis

**Figure 3:**
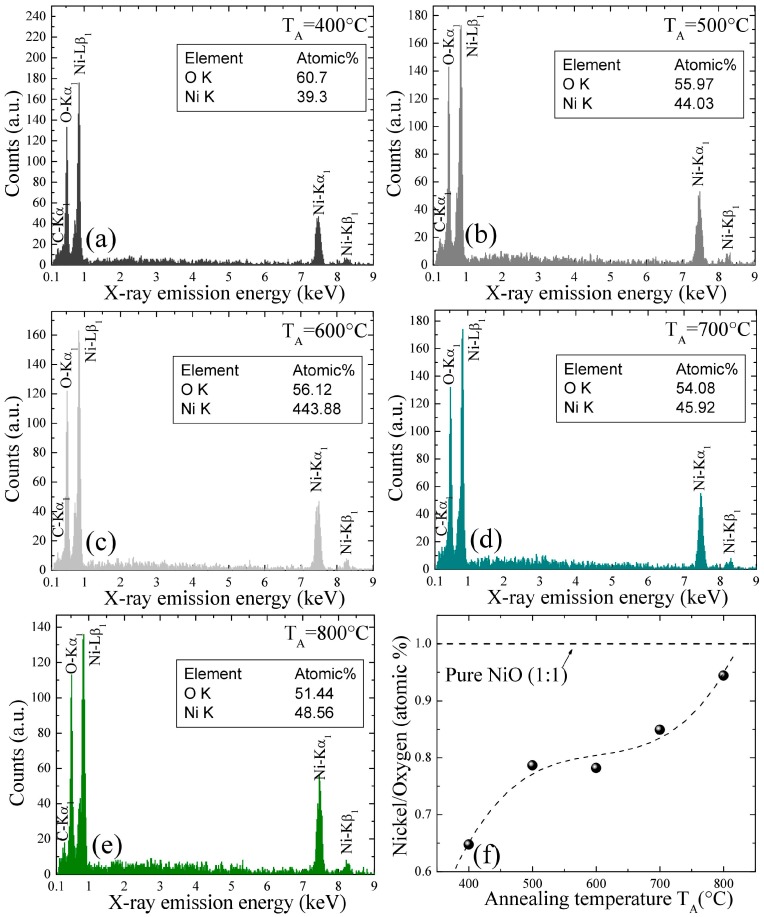
(**a**–**e**) Plots of typical EDS spectra taken from various NiO samples; (**f**) the Ni/O atomic percentage ratio with respect to annealing temperature obtained using EDS.

The NiO nanoparticles, as synthesized through chemical means, exhibited an evolution of color from black to green with an increase in the annealing temperature, following the same trend as the mean size of the nanoparticles. Small NiO particles (<20 nm) were black. The change in color from green to black for NiO nanoparticles is attributed to the presence of Ni vacancies (point defects). Energy-dispersive spectroscopy (EDS) is a useful technique for estimating the atomic percentages of constituent elements in a given sample. Figure 3a–e presents the EDS results, showing that the elemental spectra of different samples are associated with a series of elemental nickel and oxygen constituents that can be assigned to Ni-L*_β_*_1_, Ni-K*_α_*_1_, Ni-K*_β_*_1_, and O-K*_α_*_1_. The small, intense peak of C is the result of a carbon film as a consequence of mounting the sample. Besides nickel, oxygen, and carbon, no other elements could be found. The estimated Ni/O atomic percentage ratio (obtained using EDS), as a function of the annealing temperature, is plotted in Figure 3f. An increase in the Ni/O atomic percentage ratio by increasing the annealing temperature (i.e., increasing the nanoparticle size) was observed. Based on the increase of the Ni/O ratio (less than 1) in the range of T_A_ = 400–500 °C, it is likely that the formation proceeds through the nucleation of NiO and nickel hydroxide (Ni(OH)_2_) during the annealing process, which is in agreement with previous results [21]. It was observed that β-phase Ni(OH)_2_ is completely converted to NiO after being heated at 600 °C for 2 h. The colors of the powder samples and EDS spectra confirm that the stoichiometry of chemically synthesized NiO nanoparticles decreases with decreasing particle size and that small nanoparticles (i.e., up to 14(3) nm) are highly non-stoichiometric.

As discussed in our previous work the vibrational properties, i.e., phonon modes, are highly sensitive to bond strength, determined by the electron density and distribution in the lattice arrangements [21]. Therefore, it is clear that a redistribution of electron density between cations and anions will influence the physical properties of NiO nanoparticles. Among them, spectroscopic investigation could provide valuable information that can be directly correlated to the formation of lattice defects and changes in electron density due to surface nickel vacancies.

### 3.3. Photoluminescence

**Figure 4:**
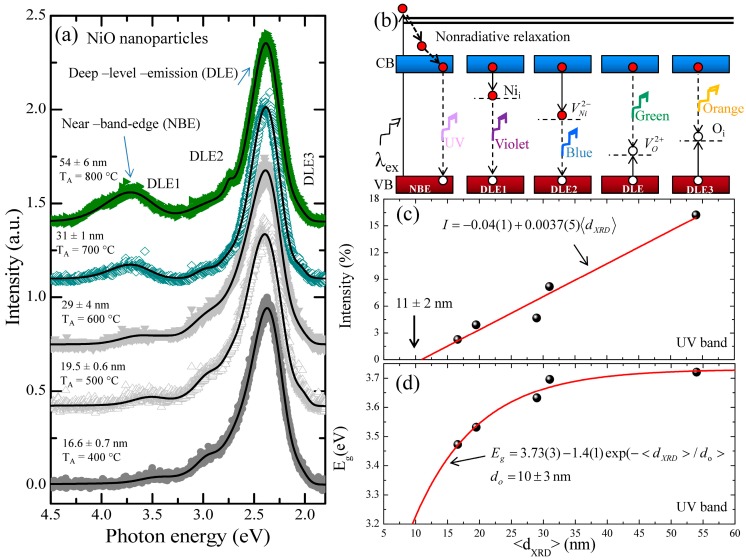
(**a**) Crystallite size dependency of room-temperature PL spectra, revealing narrow band gap emission extending from the UV to the visible region, where the solid line represents fitted PL spectra using Voigt function; (**b**) demonstrating various PL emission components that originated due to electronic transition between different defect levels and band edge of NiO nanoparticles. Crystallite nanoparticle size dependencies of (**c**) intensity of UV emission (**d**) band gap energy E_g_. The solid lines represent a linear fit to the intensity data and an exponential fit to the emission energy, respectively.

Figure 4a shows the effect of annealing temperatures from 400 °C to 800 °C (bottom to top) on normalized PL spectra of all NiO nanoparticles excited by using 266 nm (~4.66 eV) laser. In pure, stoichiometric NiO nanoparticles synthesized using the microwave combustion method, only emissions related to UV band at 346 nm (~3.58 eV) have been reported [14]. The observed wide range of emissions bands from this set of nanoparticles, extending from the UV to the visible region, confirmed that NiO nanoparticles are highly defective. The PL spectra from all these nanoparticles is dominated by the defect-related, deep-level-emission (DLE) green luminescence at ~520 nm (~2.38 eV), accompanied by a relatively weak and broad near-band-edge (NBE) UV emission at 333–357 nm (3.47–3.72 eV). The UV emission originated from excitonic recombination corresponding to NBE transition of NiO [13], while DLE in the visible region, designated as DLE1 to DLE3, are due to various structural defects such as oxygen vacancies and interstitial defects. The PL spectra in Figure 4a can be deconvoluted using Voigt distribution functions, as shown by the solid line [33]. The corresponding fitting parameters for PL spectra of all the nanoparticles are summarized in Table 2. After deconvolution of PL spectra of 16.6 ± 0.7 nm nanoparticles, five peaks have been categorized as the UV spectrum at 357 ± 1 nm (~3.47 eV), violet at 417 ± 1 nm (~2.97 eV), blue at 459 ± 1 nm (~2.70 eV), green at 523.6 ± 0.3 nm (~2.37 eV), and orange at 604 ± 1 nm (~2.05 eV), respectively. A schematic plot of various PL emission components originating due to electronic transition between different defect levels and the band edge of NiO nanoparticles is given in Figure 4b. A relatively weak and narrow UV emission band is associated with the excitonic recombination of electrons in the conduction band (CB) and holes in the valence band (VB) corresponding to the NBE transition. The violet luminescence peak (DLE1) at 417 ± 1 nm (~2.97 eV) appeared through the possible transition of trapped electrons at Ni interstitial (Ni_i_) to the valence band. The blue luminescence peak (DLE2) at 459 ± 1 nm (~2.70 eV) appeared through radiative recombination of electrons from the doubly ionized Ni vacancy ($V_{Ni}^{2-}$) to the holes in the valence band. The intense green band (DLE) at 523.6 ± 0.3 nm (~2.37 eV) is attributed to nickel vacancies [16]; however, the possibility of origin of green-band emission due to oxygen vacancies cannot be ruled out [12]. Figure 4c shows the variation in intensity of UV band with respect to crystallite size. The solid line represents a linear fit of the data, having a slope of 0.0037 ± 0.0005/nm. Extrapolating linear fit to zero intensity gives a critical crystallite size of 11 ± 2 nm, below which, due to a high surface nickel vacancy concentration, the UV emission will disappear. The similar critical size of ~11 nm has also been predicted from the intensity of the TO phonon mode in our previous report [21]. Figure 4d depicts the exponential dependency of UV emission band gap energy on the crystallite size where the solid line is an exponential decay fit to the data. The fitting parameter yields a critical crystallite size of 10 ± 3 nm with a critical band gap energy of 3.22 eV. When the crystallite size increases, the energy of the UV emission band increases and is saturated at 3.73 ± 0.03 eV, which is close to the NBE value corresponding to bulk NiO, varying between 3.6 to 4.0 eV [34,35,36,37]. The suppressed UV band emission energy observed with the decrease of crystallite size can be attributed to the weakened covalent Ni–Ni and ionic Ni–O bonds, as observed from electron density analysis. Kisan et al. [13] reported a similar value of band gap energy, ~3.45 eV (for 16.6 ± 0.7 nm, E_g_ ~ 3.47 meV) but with enhanced intensity for ~15 ± 1 nm size of stoichiometric NiO nanoparticles without any emission in the visible region. In their work further reduction of crystallite size down to ~10 nm showed a blue shift in UV emission due to the quantum confinement effect. The observed phenomenon in this set of NiO nanoparticles contradicts the quantum confinement effect observed in stoichiometric NiO nanoparticles. Furthermore, in large particles, the emission energy of blue emission band shifts to higher energy, 451.3 ± 0.2 nm (~2.75 eV), whereas the violet emission band shifts to lower energy, 430 ± 2 nm (~2.88 eV). An increase in crystallite size to 54 ± 6 nm resulted in a relatively intense UV band with a band gap energy shift to 333.2 ± 0.2 nm (~3.72 eV). NiO has a complex band structure with multiple valence (3d band of Ni^2+^ and 2p band of O^2−^) and conduction (4s band of Ni^2+^ and 3s band of O^2−^) bands. In general, the stronger the band–band PL intensity of NiO nanoparticles, the higher the recombination rate of the photoinduced electron-hole pair. The deeply trapped holes are more or less localized, exhibiting a lower oxidizing potential [38,39]. Thermal annealing treatment may result in a slight deviation from NiO stoichiometry and the cation vacancy and/or interstitial oxygen trapping in the NiO lattice leads to a main DLE peak at 523.6 ± 0.3 nm (~2.37 eV) in the green emission band that confirmed the presence of such defects in the NiO lattice. Nickel vacancies can be produced due to the charge transfer between Ni^2+^and Ni^3+^ [40]. The PL intensity of broad near-band-edge (NBE) UV emission at 333–357 nm (3.47–3.72 eV) is enhanced with an increase in the electron density, as seen in Figure 2b, because of the increase in the crystallite size. Therefore, small particles having high nickel vacancies on the surface (that is, a low electron population in the valence band) when excited will result in reduced intensity due to a suppressed electron hole recombination process. On the other hand, in thermal annealing processes, enhanced intensity UV band emission will be observed in large particles due to an increased electron hole recombination process.

**Figure 5:**
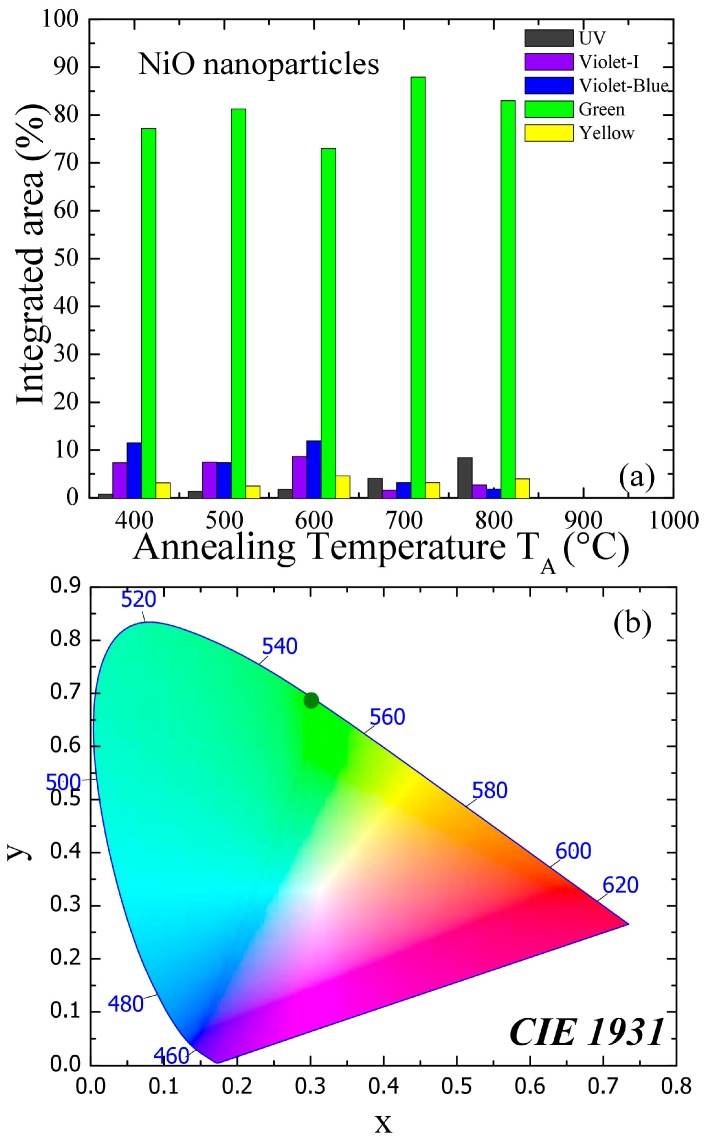
(**a**) Plot of crystallite size dependence of integrated intensity of the UV, violet, violet-blue, green, and yellow emission band, respectively; (**b**) the CIE chromaticity diagram of NiO nanoparticles.

As shown in Figure 5a, the integrated intensity of the green emission band occupies an almost constant value of ~80 % of PL spectra from all the samples, suggesting that the annealing conditions favor their formation. On the contrary, the UV emission band is strengthened and shows a linear dependency on crystallite size. Similarly, the integrated intensity of violet and blue band emission shows a constant value of ~8 and ~10%, respectively, up to 29 ± 4 nm, above which it reduces drastically to ~2% from 54 ± 6 nm nanoparticles. The former emission band is sensitive to interstitial nickel and later to nickel vacancy defects. Similarly, if the green emission band is originating due to nickel vacancies, its intensity should depend largely on the concentration of nickel vacancies in the nanoparticles; however, that is not the case in this set of nanoparticles. During the annealing process in the ambient atmosphere, it is more favorable to generate a vacancy than interstitial defects, if an energy and chemical balance between the nanoparticles and the ambient gas is considered. Therefore, oxygen and nickel vacancies can be formed simultaneously and could be the most probable candidates for the green emission band. However, observed suppressed integrated intensity of blue and violet emission band in large particles clearly indicates that oxygen vacancy could be the cause of the intense green band emission. Therefore, green band emission spectra around 523.6 ± 0.3 nm (~2.37 eV) arise when the photogenerated holes trapped in the deep level oxygen vacancy ($V_{o}^{2+}$) recombine with the electrons trapped at a shallow level located just below the conducting band. It is worth noting that the green band emission due to oxygen vacancies occurs at a slightly lower energy of 2.37 eV than the reported value of 2.48 eV due to nickel vacancies [16]. A similar effect from zinc and oxygen vacancies on green band emission in ZnO nanostructures has been reported by Børseth et al. [24]. They observed green band emission at 2.35 ± 0.05 eV by zinc vacancies and at 2.53 ± 0.05 eV by oxygen vacancies. Furthermore, due to annealing, dehydration takes place and therefore the observed orange band emission appearing at 604 ± 1 nm (~2.05 eV) is due to electron transition from the conduction band to interstitial oxygen, O_i_ and the related defect is the acceptor responsible. The integrated intensity of the orange band occupies only 4% of the PL spectra and does not change with crystallite size, indicating the presence of a small amount of interstitial oxygen in all the samples. Figure 5b shows the CIE 1931 color space chromaticity diagram in the (*x*, *y*) coordinates system. The chromaticity coordinates are (0.3012, 0.6877), (0.3011, 0.6850), (0.3011, 0.6862), (0.3012, 0.6875), and (0.3010, 0.6872), with correlated color temperatures (CCTs) of 5953, 5957, 5956, 5953, and 5956 K for nanoparticles with crystallite size varying from 19.5 ± 0.6 nm to 54 ± 6 nm, respectively. A color space chromaticity diagram shows the green color of the PL emissions from the variously sized NiO nanoparticles. Although the PL spectra in Figure 4a consist of varying visible light emissions, from violet to orange, they were mainly (~80%) comprised of green light emissions, as shown in Figure 5a. The NiO nanoparticles have effective green band emissions at room temperature, so they all are potential candidates for use in optoelectronic nanodevices, such as light-emitting diodes and laser diodes in green band emission.

## 4. Conclusions

Room-temperature photoluminescence spectroscopy was successfully utilized to study the effect of surface nickel vacancies on the photoluminescence of NiO nanoparticles. The emission in UV region is attributed to excitonic recombination of electron and holes corresponding to the near-band-edge transition. The obtained value of UV emission band decreases when decreasing the particle size from 54 ± 6 nm (3.72 eV) to 16.6 ± 0.7 nm (3.47 eV), accompanied by the weakening of ionic Ni–O and covalent Ni–Ni bonds determined by the electron density distribution calculation, hinting at a possible way to tune the Ni–O and covalent Ni–Ni bond strength to control the variation of the UV emission band in a nanostructure system. The green band emission spectra around 523.6 ± 0.3 nm (~2.37 eV) arises when the photogenerated holes trapped in the deep level oxygen vacancy ($V_{o}^{2+}$) recombine with the electrons trapped at a shallow level located just below the conducting band. This apparently anomalous phenomenon in visible region weak violet, blue, orange, and strong green band emissions results from the related defects and can be attributed to interstitial nickel, nickel vacancies, interstitial oxygen, and oxygen vacancies, respectively, in the deep level emission.

## Figures and Tables

**Table 1:** Peak charge density along the bonding directions of NiO (*d*: distance, *ρ*_e_: electron density).

Bond Critical Point					Peak Charge Density					
T_A_ (°C)	Ni–O		O–O		Ni–O				O–O	
					Ni		O		O	
	*d*(Å)	*ρ*_e_(e/Å^3^)	*d*(Å)	*ρ*_e_(e/Å^3^)	*d*(Å)	*ρ*_e_(e/Å^3^)	*d*(Å)	*ρ*_e_(e/Å^3^)	*d*(Å)	*ρ*_e_(e/Å^3^)
400	1.0914	0.4957	1.4841	0.1728	0	164.386	2.0989	18.8958	2.9623	18.8958
500	1.0910	0.5189	1.4835	0.1766	0	148.289	2.0983	16.7023	2.9615	16.7023
600	1.0906	0.5667	1.4829	0.1912	0	105.241	2.0979	15.7964	2.9609	15.7964
700	1.0900	0.5648	1.4823	0.1939	0	127.527	2.0954	15.8163	2.9574	15.8163
800	1.0896	0.5744	1.4817	0.1939	0	126.492	2.0951	15.2787	2.9574	15.2787

**Table 2:** Summary of Voigt distribution function fitting parameters for PL spectra of NiO nanoparticles.

Mean Size (nm)	UV Region (nm)		Visible Region (nm)							
	NBE	FWHM	DLE1	FWHM	DLE2	FWHM	DLE	FWHM	DLE3	FWHM
16.6 ± 0.7	357 ± 1	35	417 ± 1	48	459 ± 1	60	523.6 ± 0.3	78	604 ± 1	50
19.5 ± 0.6	351 ± 1	36	418 ± 1	46	451 ± 1	48	518.4 ± 0.3	85	611 ± 1	43
29 ± 4	356 ± 2	39	420 ± 5	57	468 ± 4	58	521 ± 3	75	598 ± 2	59
31 ± 1	335.4 ± 0.1	40	415 ± 1	28	448 ± 1	36	520 ± 1	73	605 ± 1	42
54 ± 6	333.2 ± 0.2	48	430 ± 2	25	451.3 ± 0.2	16	520.2 ± 0.1	72	595 ± 1	55
